# Patients’ Experiences of Using a Smartphone App After Cardiac Rehabilitation: Qualitative Study

**DOI:** 10.2196/34294

**Published:** 2022-03-23

**Authors:** Pernille Lunde, Asta Bye, Kari Anette Bruusgaard, Elisabet Hellem, Birgitta Blakstad Nilsson

**Affiliations:** 1 Department of Physiotherapy Faculty of Health Sciences Oslo Metropolitan University Oslo Norway; 2 Department of Nursing and Health Promotion Faculty of Health Sciences Oslo Metropolitan University Oslo Norway; 3 European Palliative Care Research Centre Department of Oncology Oslo University Hospital Oslo Norway; 4 Section for Physiotherapy Division of Medicine Oslo University Hospital Oslo Norway

**Keywords:** mHealth, mobile health, cardiac rehabilitation, mobile phone app, smartphone, lifestyle

## Abstract

**Background:**

Exercise-based cardiac rehabilitation (CR) is a crucial part of the treatment of patients with cardiac diseases, and adherence to healthy behavior is a prerequisite to improve long-term prognosis. Unfortunately, adherence to healthy behavior adapted in CR is challenging for many cardiac patients in the long term. Recently, we demonstrated that follow-up conducted via an app for 1 year significantly improved adherence to healthy behavior after CR. To increase the knowledge and understanding of mobile Health (mHealth) interventions that can promote acceptance and adherence, qualitative research investigating patients’ experiences with these interventions is warranted.

**Objective:**

The aim was to investigate patient experiences with individualized long-term follow-up conducted via an app for 1 year and their thoughts about what features promoted adherence to healthy behavior after CR. The purpose was to increase the understanding of significant findings previously reported and to guide future development of similar interventions in the field of adherence.

**Methods:**

A qualitative study with individual interviews was conducted from November 2018 to May 2019. A thematic interview guide was used when conducting the semistructured in-depth interviews. The interviews were audio recorded and transcribed successively during the period in which the interviews were conducted. Texts were managed and systematized by NVivo. Interviews were analyzed by qualitative content analysis. Codes and themes were inductively developed.

**Results:**

Ten patients who had participated in a randomized controlled trial evaluating the effect of follow-up conducted via an app on adherence to healthy behavior after CR were included. The median patient age was 65 years (range 46-72 years), and both genders were represented. The analysis resulted in the following 4 themes describing the patients’ experiences: (1) The person behind the app is crucial for motivation and adherence; (2) The app as a commitment; (3) The app as a path to independence; and (4) Suggestions for improvements. Features experienced as beneficial to promote adherence were individualized feedback and the use of goal setting. The significance of the person behind the app (the supervisor) who provided individualized feedback was a consistent finding. This person seemed to promote motivation in general and to enable other known behavioral change techniques.

**Conclusions:**

The person behind the app (the supervisor) seems to be one of the most significant success factors in promoting adherence to healthy behavior after CR. This indicates that a health care provider must actively participate in a patient’s process of adherence to healthy behavior, even when using interventions, including an app. Future development of interventions in the field of adherence should strive to create tools that enable an ongoing collaborative relationship between the patient and the health care provider. The follow-up should be based on the patient’s own goals, and individualized feedback should be provided.

## Introduction

Exercise-based cardiac rehabilitation (CR) is a crucial part of the treatment of patients with cardiac diseases and is a Class IA recommendation in European guidelines [1,2]. The overall goal of secondary prevention, including CR, is to prevent subsequent cardiac events [2,3]. In this context, adherence to healthy behavior, including physical activity, regular exercise, healthy nutrition with bodyweight control, compliance with taking medication, and smoking cessation [2], is crucial. Although adherence to healthy behavior is a prerequisite to improve the long-term prognosis, the majority of cardiac patients do not achieve the guideline standard for secondary prevention in the long term [2,4]. Research evaluating interventions aiming to improve adherence to healthy behavior after CR is therefore warranted [2].

Mobile health (mHealth) interventions have been proposed to meet the challenges related to adherence to healthy behavior and have thus been suggested as potential interventions after CR [2,5-7]. In particular, smartphone apps have been considered promising owing to their ability to monitor patients’ health from anywhere at any time [5,8]. Previous research has highlighted the need for individualization of such interventions [9,10]. Recently, we demonstrated the feasibility of using an app to provide individualized follow-up in patients after CR [11]. Based on the results from this study, we developed and conducted a randomized controlled trial (RCT) aiming to evaluate the effect of individualized follow-up with an app for 1 year on health outcomes relevant for adherence to healthy behavior in patients after CR [12]. Patients in the intervention group received access to an app where they added individual goals and accompanying tasks [12,13]. They were monitored and followed by a supervisor (specialized physiotherapist) for a year. The app itself provided reminders and evaluations of tasks and weekly goal achievement, and the patients could write notes related to each goal. The intervention included comprehensive individualized feedback, based on the patients’ goals and what they had done, through an email every week for the first 12 weeks and every fourth week for the rest of the year. Throughout the year, they also received between 1 and 3 short motivational messages every week. These messages were written individually for each patient. However, sometimes the content was of a more general nature. Additionally, patients could submit questions to the supervisor and receive answers within 2 working days throughout the year [12,13]. The results demonstrated that using the app significantly improved peak oxygen uptake, exercise performance, exercise habits, and self-perceived goal achievement, compared with a control group that received usual care after CR [12]. All patients allocated to the intervention group used the app, and as much as 71% of the patients used the app on a daily or weekly basis throughout the year [12].

The high acceptance and use of the app in our study was unique as difficulty or low acceptance in using the technology is a frequent obstacle in similar interventions [14]. In order to increase the knowledge and understanding of mHealth functions and components that can promote acceptance and adherence, qualitative research investigating patients’ experiences with these interventions is urged. To our knowledge, no previous studies have explored patients’ experiences with individualized mHealth interventions lasting for a whole year. The purpose of this study was to increase the understanding of the significant findings previously reported [12] and to guide future development of similar interventions in the field of adherence. Our aim was to investigate patient experiences with individualized long-term follow-up conducted via an app for 1 year, in order to gain more knowledge about features promoting adherence to healthy behavior after CR.

## Methods

### Design

A qualitative study with individual interviews was conducted to describe patients’ experiences with a long-term follow-up intervention conducted via an app. The interviews were planned to be completed within 2 weeks after the patients had ended their follow-up period of 1 year in the previously mentioned RCT [12].

### Recruitment

Participants were recruited from the RCT [12] (n=113). Enrollment in the RCT was carried out at 2 CR centers in the eastern part of Norway from October 2017 to June 2018. These CR centers offered, in total, 3 different CR programs: 12-week outpatient CR, 4-week inpatient CR, and 1-week inpatient CR. The randomization was stratified by the CR program to ensure equal participation and thereby representativeness.

At the 1-year follow-up assessment, participants in the intervention group were recruited in this study. Living nearby Oslo (maximum 1-hour commute) was set as an inclusion criterion as the interviews were planned to be conducted at Oslo Metropolitan University (OsloMet) in Oslo, Norway. Efforts were made to ensure that participants were representative of the CR population in the eastern part of Norway (both genders, participation in different CR programs, and different ages). Recruitment and inclusion in this study continued until data saturation was achieved.

### Interviews and Interview Guide

Individual interviews were completed from November 2018 to May 2019. The interviews were carried out at OsloMet. One participant chose to complete the interview digitally owing to several unforeseen appointments that made it difficult to attend physically. To ensure sufficient quality on the audio recording, Skype for Business was used. The interviews lasted from 35 to 62 minutes (44 minutes on average) and were carried out by 2 researchers (KAB and EH) who did not take part in the RCT from which the participants were recruited. Both interviewers had extensive experience from CR and qualitative research. To ensure the material was as comprehensive as possible, all interviews were carried out with both researchers present.

A thematic interview guide (Multimedia Appendix 1) was used when conducting the semistructured in-depth interviews. The interview guide was developed by 3 of the authors (PL, KAB, and EH) and validated by all authors. The aim was to maintain an open nonjudgmental attitude. Emphasis was placed on listening to the responses to open-ended questions and allowing the participants to fully explain a phenomenon, together with an invitation to reflect upon their experiences [15]. The interviews were audio recorded and transcribed successively during the period in which the interviews were conducted. Initially, the texts were managed and systematized in Microsoft Word, and by working manually with printouts and pen and paper. Thereafter, the texts were imported to, and managed and systematized by NVivo (released in March 2020) [16]. Quotations from the texts were translated from Norwegian to English by the first author (PL) and then validated by all co-authors.

### Data Analysis

Transcribed interviews were analyzed by a thematic coding technique based on the framework by Braun and Clarke [17], which is a method for identifying, analyzing, and reporting patterns within qualitative data. The method includes the following 6 phases: (1) familiarization with the data, (2) generating initial codes, (3) searching for themes, (4) reviewing themes, (5) defining and naming themes, and (6) writing the report [17]. The codes and themes were inductively developed.

Initially, the analysis involved repeated readings of each transcript by all the authors to obtain an overall impression of the material. The next phase involved coding the entire data set on a semantic level. Specifically, we focused on the parts of the data that revealed relevant information and descriptions regarding the current overall research question. Further, codes that revealed similar aspects of the data were grouped into preliminary themes, which were checked for consistency and variability within and across interviews. Subsequently, we identified and interpreted 4 overarching themes in a constant process of moving between the data, potential themes, and maps made for visualization, as well as in reference to relevant literature, and discussions and mutual understanding among the authors. Finally, themes were established if they were coherent and represented the meanings found in the interviews [17]. Throughout the analytic process, all findings were discussed and validated within the research group. In case of inconsistencies, further discussions and reflections were used for resolution.

### Ethical Considerations

This study was approved by the Regional Committee for Medical and Health Research Ethics (South-East ID: 2016-1476) as a substudy of the previously described RCT. All included patients provided written informed consent.

## Results

### General Findings

Ten patients with a median age of 65 years (range 46-72 years) participated in the study (Table 1). More than half of the patients were retired. The majority had participated in a 4-week inpatient CR program or a 12-week outpatient CR program before inclusion in the RCT. All patients had their own goals or tasks related to exercise and physical activity. Additionally, 7 of the patients had goals related to weight loss or maintenance of bodyweight, and accompanying tasks were specific nutritional advice learned or implemented in primary CR. The numbers and types of goals are presented in Table 1. Nine of the patients attended the interview as scheduled, while 1 was unable to attend until 4 weeks after the follow-up assessment, due to other medical and social appointments.

All patients in the study mentioned that they used the app for preventive activities, such as exercise, physical activity, and healthy nutrition, and, without exception, they found the app easy to use. The patients’ experiences evolved within the following 4 themes: (1) The person behind the app is crucial for motivation and adherence; (2) The app as a commitment; (3) The app as a path to independence; and (4) Suggestions for improvements. The first overarching theme was abstracted to subthemes.

**Table 1:** Characteristics of the patients (N=10).

Characteristic		Value, n (%)
**Gender**		
	Male	9 (90)
	Female	1 (10)
**Age distribution (years)**		
	40-49	1 (10)
	50-59	2 (20)
	60-69	5 (50)
	70-79	2 (20)
**Civil status**		
	Married/cohabiting	8 (80)
	Single	2 (20)
**Employment status**		
	Employed	3 (30)
	Retired	6 (60)
	Disability benefits	1 (10)
**Disease**		
	Coronary artery disease	7 (70)
	Valve surgery	3 (30)
**Type of cardiac rehabilitation**		
	One week	1 (10)
	Four weeks	4 (40)
	Twelve weeks	5 (50)
**Smartphone**		
	iPhone	7 (70)
	Android	3 (30)
**Number of goals**		
	One	4 (40)
	Two	6 (60)
**Type of goal**		
	Exercise-related goal	9 (90)
	Weight loss/maintenance goal	7 (70)

### The Person Behind the App is Crucial for Motivation and Adherence

All patients in the study highlighted that the person behind the app (the supervisor) was considered a prerequisite to succeed with the intervention. However, this person cannot be just anyone. The patients highlighted that the person must possess a set of characteristics that primarily helps create a relationship of trust between the supervisor and the patient, which helps to make the app motivating and thereby helps the patient adhere to healthy behavior. Personal characteristics of special importance included engagement, professional competence, care, and support.

You know, she is not just anyone, the fact is that she gets involved and shows care and engagement in me as a person. At least I perceive it as if she wants my best, and she gives the advice that is for my best.

Participant #1

It is about the individual behind it, from whom you can almost experience a kind of love, and a person who is engaged in you.

Participant #10

The following 3 subthemes evolved from this theme: (1) individualized feedback, (2) follow-up based on own goals, and (3) a lifebuoy in the event of unforeseen events. The person behind the app was the common denominator for all 3 subthemes.

#### Individualized Feedback

An important motivating factor, which was highlighted by all patients in this study, was the individual feedback that each one received throughout the study period. The person behind the app made it possible to provide tailored feedback, advice, and guidance, which seems to have been a success criterion. The tailoring should be based on the patients’ individual condition, the recent development, and the patients’ likes and dislikes. This reinforces the feeling that the feedback is directed at the individual and not in general. Several of the patients used other general health apps during the intervention period. They pointed out the difference between individual feedback and automated feedback.

I do not really believe in apps providing feedback automatically. So, this app is great because there is a person providing the feedback, which means that the feedback is directed solely to you. That is, I think that is crucial, because this is what’s motivates me.

Participant #8

The fact that there was a physical person, that you actually knew at the other end, who provided individualized feedback and you had the opportunity to communicate with, was extra motivating. This made it easier to keep up the good work.

Participant #7

#### Follow-up Based on Own Goals

The person behind the app enabled the follow-up to be based on individualized goals, which most patients highlighted as important to increase motivation.

I think that’s pretty essential (setting your own goals). Of course, the more you personify this, the better it is. And of course, following them then. However, those goals could have been more nuanced. Maybe there could have been a few more.

Participant #3

#### A Lifebuoy in the Event of Unforeseen Events

Some of the patients experienced dramatic events that caused a significant setback during the year of follow-up. They expressed that for them, most likely, the app and follow-up had been extra important for long-term adherence to healthy behavior. In these cases, the patients mentioned that it was absolutely crucial that there was a person behind the app with whom they had an established and trustful relationship.

If I didn’t have the app, or should I say “her”….. If I didn’t have her at that time, I think I would have had extensive challenges getting to where I am today…, so fast... I would probably have walked and strolled a bit, but I would not have been able to physically be where I am today. Because of that (setback), I needed help in a proper way… not like “you have to do this, and you have to do that.” …But something motivating and encouraging, and that is exactly what I got from her.

Participant #4

Additionally, adjustments and flexibility in goal-setting processes and the accompanying tasks were highlighted as central. This seems to be particularly important following dramatic events, when patients often must take one day at a time.

For me it has certainly had an extra great significance, because it was a bit like a crisis, and she came out with suggestions for alternatives to the goals I had set myself.

Participant #1

### The App as a Commitment

Several of the patients described that the app, and the follow-up, provided a form of commitment. The commitment to the person behind the app turned out to be the most evident.

To be honest, I did not want to disappoint the supervisor, because she had been so motivating. So, my wife said, “It doesn’t matter what I say, but when she says it, then it is important.” So, maybe there`s something in it.

Participant #4

Several of the patients also expressed a commitment to the research project as a motivating factor. However, the distinction between the research project and the person behind the app was not clear.

We also knew that we were part of a research project, so you kind of felt it was a bit important what you were doing. Or at least, it could make a difference to her work. That she was involved, and that it was fun to try to take it seriously…. and then, the idea with that app and the follow-up was that you should perform at your best level …. that was a good motivation.

Participant #10

Finally, the patients expressed that the app also gave a commitment to themselves. To be challenged at their individual level was highlighted as motivating. Some patients described that they used the note function in the app and wrote a diary to give themselves an extra challenge, beyond the one they received from the supervisor.

I posted such a summary, that this week I have completed 4×4 intervals, while this week I have had pyramid intervals […]. So, it was a small summary for each week, and I really appreciated it because it was very nice to be able to scroll through, and it gave a motivation to keep up the good work and to challenge myself. It also gave me bad conscience if I did not exercise enough.

Participant #7

### The App as a Path to Independence

The patients expressed that they experienced the downward adjustment regarding frequency of comprehensive feedback, also known as individualized feedback, as overwhelming and a bit scary. Despite this, the downgrading was perceived as important to increase independence while they at the same time felt safe and supported on a regular basis. Additionally, they knew they could easily get in touch with the supervisor if needed.

Right away it was a little shocking, like “Oh? Is it only once a month, now?” It was so nice with that attention…. But then, sort of, yes, that was the deal. I have reached a higher level…. Now, I must be more independent. […] I must keep it going by myself, so in that sense it gives a natural transition. But I felt like I had been living in a suite, a first-class suite, and then suddenly, I was down to third class, sort of.

Participant #10

Most of the patients expressed that the feeling of safety that the app gave them was important to promote and push themselves to the activities that they needed to reach their goals. In particular, when the frequency of the comprehensive feedback was downgraded, this safety was extra important. Through the 9 months with less frequent follow-up, they got the chance to experience that they were able to adhere to their program almost by themselves.

So, at that time when the frequency was downgraded, I was a bit alone. However, with that app and follow-up, you have a direct link to the expertise in a way, which is both reassuring and motivating. […] It was a very good safety net, it`s like wearing a parachute. You don’t have to use it, but you know it`s there.

Participant #7

### Suggestions for Improvements

Despite the promising result in the RCT regarding the effect of follow-up with the app, we also analyzed the qualitative data to illuminate the potential for improvements to optimize future interventions in the field of adherence. Overall, patients expressed high satisfaction with the app and justified this with the fact that it was easy to use. Most of the patients considered the app to be a tool, enabling human interaction.

So, technology can’t replace people, but it is a helpful tool

Participant #1

Nevertheless, 2 suggestions for improvements clearly evolved. This was related to ownership of own goals and self-perceived goal achievement. Although most of the patients found it both meaningful and motivating that the use of the app and the follow-up were based on their own goals, ownership to some of the patients’ goals could be questioned. Some of the patients expressed that their goals were made by health care providers at the CR center before completing CR. As a result, they did not necessarily consider the goals to be their own. Additionally, the opportunity to change goals along the way was raised as a potential improvement. Unforeseen events may occur at any time, which may affect the possibility of achievement and ownership of previously set goals. This demands greater flexibility in goal setting throughout the year. Finally, in the RCT, patients in the intervention group were asked to rate self-perceived goal achievement on a Likert scale (0-100) weekly [12,13]. All patients in this study expressed that this question was difficult to answer, and several of the patients described the scale and question as abstract.

## Discussion

### Principal Findings

Our findings indicate that a supervisor who possesses special characteristics is crucial to receive the full benefit of an app for increasing adherence to healthy behavior after CR. Confidence in the supervisor seems to be what enables other highlighted functions and components of the app to be perceived as motivating in relation to adherence. Other features of the app highlighted by the patients were that the app made it possible to provide individualized feedback and the use of the app was based on own goals. Additionally, the app provided a form of commitment, which proved to be of importance. Finally, to succeed in the hard work of adherence to healthy behavior after CR, patients highlighted the importance of gradually phasing out the follow-up and feedback from the supervisor.

All patients in this study highlighted the importance of the person behind the app. They described how their experiences with the supervisor’s engagement, care, and support, as well as professional competence promoted motivation to adhere to healthy behavior. The trust-based relationship between the patient and supervisor could be considered a prerequisite for other components of the intervention promoting motivation to adhere to healthy behavior. To our knowledge, no qualitative studies evaluating patients’ experiences using apps have clearly stated the essence of the person behind the technology. On the other hand, this finding is not surprising, as a concept analysis of adherence in the context of cardiovascular risk reduction states that adherence implies active participation and collaboration and is dependent on a concordant relationship between the patient and the health care provider [18]. A trustful relationship with a health care provider has been considered crucial for establishing strong adherence to healthy behavior [18]. An ongoing collaborative relationship between the patient and the health care provider is considered one of the most important attributes of successful adherence [19].

In this study, all patients experienced the feedback as particularly meaningful because it was individually tailored. Individual tailoring demands a person behind the app who administers the feedback. Feedback has been emphasized in the framework for the development of mobile technology use in CR [5]. In particular, individualized feedback has been proposed as a superior technique for long-term success [5,20], and may reflect the attributes of ongoing support and collaboration with a health care provider [18]. It may also reflect the supervisor’s ability to influence the patients’ self-efficacy [21]. People with high self-efficacy are more likely to believe that they can change their behavior than people with low self-efficacy. A positive association between self-efficacy and adherence to exercise has been described in people with coronary heart disease [22]. This is in line with a narrative review that states the importance of self-efficacy in exercise adherence among patients with chronic heart failure [23]. Exercise and exercise-based CR, which improve physical function, seem to be beneficial in order to increase self-efficacy in exercise adherence [23]. We also believe that a prerequisite for the supervisor to succeed in strengthening the patient’s self-efficacy using an app is patient participation in an exercise-based CR program prior to the follow-up with the app, as in our study. Patients in our RCT were recruited from exercise-based CR programs. One of the centers documented significant improvement in peak oxygen uptake after a 12-week CR program [24], which is likely a great booster of self-efficacy.

Another factor mentioned by most patients as important for generating motivation was that the app and the follow-up provided a commitment. The commitment was 3 fold, where the commitment to the person behind the app seemed to be the strongest. However, commitment to oneself also evolved as an important factor. The possibility of the app to aid in self-monitoring worked as a personal challenge and was described to be of value to adherence. We believe that this finding can be understood in the light of the app providing internal motivation. Internally motivated changes are considered significant for success in adherence to long-term behavioral changes [18,25].

Another attribute of successful adherence is experiencing the achievements of one’s goals [19]. Most of the patients mentioned that it was important that the app and the follow-up were based on their own goals. Some even felt that this was essential to promote motivation. Goal setting is established as an effective technique in behavioral change, and setting specific goals has been shown to be effective for increasing patients’ levels of physical activity after CR in terms of both frequency and duration [20]. However, guidance in setting goals that are small, important for the patient, specific, and achievable is essential to succeed with the technique [26]. Even though both CR centers included in this study considered goal setting with the patient important and the supervisor was an experienced physiotherapist from CR, some patients still mentioned an absence of ownership to their goals. Goal setting seems to be of great importance, and strategies for the implementation of the process should be highlighted in future similar interventions in the field of adherence. The importance of ownership to one’s goals should not be underestimated. To maintain goals as a motivating factor for adherence to long-lasting interventions, there is a need for flexibility in terms of changing goals in line with changing needs.

The use of behavior change theory in crafting interventions has shown more powerful effects compared with interventions not based on theory [27]. The same applies to technology-based interventions. Applying behavior change theory is associated with an increased likelihood of effects in technology-based interventions [28]. The theoretical framework is important in understanding how changes are achieved [28,29]. The intervention evaluated in this study was based on the transtheoretical model (TTM) of behavior change, also known as the stages of change model [30]. According to this model, behavioral change is a process that rarely occurs in a linear manner [30]. Some of the patients experienced unforeseen events resulting in setbacks during the process toward permanent changes. They described the app and the tailored follow-up in the setback stage as a lifebuoy that helped them come back on the right track. The TTM emphasizes that setbacks in terms of moving back to a lower stage of change, that is, from the stage of maintenance to the stage of action or preparation, are more common than unusual [30]. Further, the TTM emphasizes that the need for support may be different at different stages and should be tailored to increase the likelihood of successful behavior change [30].

Interestingly, no patients suggested technical improvements of the app directly. However, many patients mentioned that the weekly rating (0-100) of self-perceived goal achievement was difficult and pointless. Therefore, a concrete improvement of the intervention would be the removal of this component. Overall, the satisfaction with the intervention, including the technical solution of the app, was high, and the use of the app was high [12]. We believe that a reason for this was that the RCT followed the Medical Research Council complex intervention framework [29,31], that is, careful and structured development of the intervention based on an evidence base and a theoretical framework [11,32,33]. A greater degree of ownership of goals was another suggestion for improvement. This will be carefully assessed and taken into account in our future planned implementation study. Additionally, we believe that a potential improvement could be the assignment of the patient’s supervisor based on the patient’s goals. For example, it can be beneficial if the supervisor is a nutritionist when the patient’s goals are primarily diet related. This was not explicitly mentioned by the patients, but is based on the fact that more than half of the patients had goals related to weight loss, and results from the RCT did not demonstrate any statistically significant effect on bodyweight [12].

It is difficult to state whether our findings are unique as few comparable studies exist. However, a recently published systematic qualitative grounded theory review aimed at investigating the barriers to and facilitators of technology in CR and self-management [34] supports our findings. Background knowledge, ongoing support, and in-the-moment understanding, as well as personalization and gamification were concluded as facilitators [34].

### Methodological Reflections and Limitations

The strength of this study is that patients from all 3 CR programs were invited to participate in the interviews, which represented the heterogeneity of patients in CR (both genders, younger and older patients, and patients living in rural and urban areas). This strengthens the credibility of the data. However, few women and few patients who originally attended the 1-week CR program were included. Their experiences are therefore represented to a lesser extent when compared with that for men and patients who originally attended the 4-week or 12-week CR program. The project leader (PL) strived to recruit more women and more patients originally from the 1-week CR program, but due to the inclusion criterion of living nearby Oslo, it was not possible. To ensure trustworthiness, all authors collaborated on the data analysis. The fact that 5 researchers conducted the analysis is expected to strengthen the dependability and overall trustworthiness. The sample size can be regarded as small, but the interviews were nuanced, and we considered the material to be saturated after 8 interviews. This view was also valid after 10 interviews when we decided to end the data collection.

The purpose of qualitative research is directed toward providing in-depth explanations and meanings rather than generalizing findings [35]. The term “transferability” is used to express to what degree the findings can be applied to other contexts. The transferability of this study has to be judged by the reader. We hope to have highlighted some phenomena that may have relevance for comparable patient populations and situations, such as app-based interventions aiming to promote adherence to healthy behavior in patients with lifestyle diseases.

Since the interviews were conducted after the end of the intervention in the RCT, oversights and recall biases of relevant experiences and suggestions for improvement cannot be ruled out. Interviews during different phases of the intervention (ie, after 3, 6, and 12 months) could have resulted in more accurate snapshots of the patients’ experiences.

Regarding the positions and preconceptions of the researchers, the first author’s first-hand experiences with the intervention through being the project coordinator and supervisor for all patients included in the RCT may have had an influence. For example, the overall idea of evaluating patients’ experiences and thereby the choice of the research question was based on regular feedback from patients during the RCT. Further, the engagement of 2 authors (AB and BBN) in the RCT, which this study builds on, may also have had an influence. Even though all the authors have professional and research interests in the field of health science, there was diversity among author backgrounds (physiotherapy and dietetics), as well as diversity in relation to author experiences with the use of technology and their previous engagement in the RCT. This led to interesting discussions and enhanced reflexivity [36]. The overall experience of the researchers of this study most likely indicates that there were certain things that we took for granted. However, it also means that we were well positioned to understand the context and to perform the study [37].

### Conclusions and Implications

Overall, appreciation of the person behind the app turned out to be a consistent finding. This person seems to promote motivation in general and seems to enable other known behavioral change techniques to be motivating, such as feedback and goal setting. Therefore, the person behind the app (the supervisor) seems to be one of the main reasons for the high acceptance and use of the app, and consequently, is important for the results in the RCT. We therefore conclude that health care providers should actively participate in the patients’ process of adherence and that the use of the app should not be considered a substitute but a reinforcement in motivational work to promote adherence to healthy behavior after CR. Future development of interventions in the field of adherence should therefore strive to create tools that enable an ongoing collaborative relationship between the patient and the health care provider; provide follow-up based on patients’ own goals, of which they have ownership; and provide feedback and support to patients at the stage of change, at any given time.

## Appendix

Multimedia Appendix 1 Thematic interview guide.

## Abbreviations

CR: cardiac rehabilitation
mHealth: mobile health
OsloMet: Oslo Metropolitan University
RCT: randomized controlled trial
TTM: transtheoretical model
