# Supplementary material for: Patients’ Experiences of Using a Smartphone App After Cardiac Rehabilitation: Qualitative Study
Source: JMIR Hum Factors. 2022 Mar 23;9(1):e34294. doi: 10.2196/34294 (PMC8987967; doi:10.2196/34294)
Supplement: Multimedia Appendix 1 [file humanfactors_v9i1e34294_app1.docx]

**Multimedia Appendix 1.** Thematic interview guide.

Background information related to health and the cardiac event

- How do you see yourself regarding your own health?
- Living heart-friendly, what do you think about that?
  - Challenges?
  - What have been difficult?
  - Why is it difficult?
- How has the cardiac event influenced you regarding challenging yourself physically?

Background information related to the use of the app

- Have you used the app?
  - Regularly?
  - For how long?
- How has it been to get acquainted with the app?
  - Did you need help? If so, from who?
- What have you used the app for?

The app`s various components

- Can you tell us a little about how important it was for you that the app and the follow-up provided was based on your personal goals?
- From a lot of different apps and other monitors you get automatic feedback based on your effort that particular day or week, as opposed to more individualized feedback.
  - What have the feedback from the supervisor meant for you?
  - What do you think about the individual feedback that you have received compared to if it had been more general and automated?
- What do you think about the frequency of the feedback?
  - Too much, too little, sufficient?
- What do you think about the level of detail in the feedback that you received?

The app in relation to lifestyle change

- How has the follow-up via the app affected what you do in everyday life? (In terms of physical activity, exercise and diet)
- How would you describe your habits (exercise, physical activity and diet) today compared to before the cardiac event?
  - How do you plan and implement physical activity and exercise these days?
  - How do you relate to a heart-friendly diet and how do you implement it to everyday life?
- Is there anything related to the app and/or the follow-up provided that has had a special impact on your interest in and knowledge of health and health promotion?
- How has the use of the app and the feedback received affected you?

Lifelong adherence to healthy behavior post-cardiac rehabilitation

- How has the app and the follow-up provided, contributed to how much you have dared to challenge yourself after completing cardiac rehabilitation?
- Now that the project is finished, and you no longer have the follow-up via the app, how do you see continuing your own?

Future implementation of using an app post-cardiac rehabilitation

- What promotes and what inhibits the use of the app?
  - Suggestions for improvements based on your needs.
- Do you have any ideas about alternative follow-up methods to promote adherence to healthy behavior after completed cardiac rehabilitation?
